# Encoding Pleasant and Unpleasant Expression of the Architectural Window Shapes: An ERP Study

**DOI:** 10.3389/fnbeh.2019.00186

**Published:** 2019-08-16

**Authors:** Parastou Naghibi Rad, Abbas Ali Shahroudi, Hamed Shabani, Sahar Ajami, Reza Lashgari

**Affiliations:** ^1^Brain Engineering Research Center, School of Cognitive Sciences, Institute for Research in Fundamental Sciences (IPM), Tehran, Iran; ^2^Faculty of Art and Architecture, University of Mazandaran, Babolsar, Iran

**Keywords:** cortical activity, EEG signals, ERP, building facades, window shapes, pleasant and unpleasant expression

## Abstract

The evaluation of building facades is one of the most important elements in built environments for helping architects and professionals to develop future designs. The form or shape of windows in building facades has direct impacts on perceivers’ affective state and emotions. To understand the impacts of geometric windows on the subject’s feedback and cortical activity, psychophysics experiments and electroencephalogram (EEG) recordings were measured from the participants. Our behavioral results show a distinguished categorization of the window shapes as pleasant and unpleasant stimuli. The rectangular, square, circular and semi-circular arch were determined as the pleasant window shapes, while the triangular and triangular arch window shapes were distinguished as unpleasant. Furthermore, event-related potential (ERP) components (N1, P2 and P3) were investigated to determine the influence of window shapes on the local brain activity. To measure reliable cortical responses, a Butterworth notch filter (50 Hz), band pass filter (0.1–60 Hz) and ADJUST filter were employed to remove the artifacts. The electrophysiological results show increased activity for the unpleasant in comparison to the pleasant windows (*p* < 0.05, Rank-Sum test) in both frontal (for P2 component) and posterio-occipital (ERP amplitudes; the N1 through to the P3 peak) channels. The ERP amplitudes of the right hemisphere were significantly larger than in the left hemisphere, not only in response to the unpleasant (*p* < 0.001) but also to the pleasant window stimuli (*p* < 0.001, Signed-Rank test). However, the unpleasant stimuli evoked significantly larger ERP amplitude than the pleasant stimuli. Moreover, the significant ERP_P2_ amplitude was more distinguished for unpleasant (*p* = 0.01, Signed-Rank test) than pleasant windows (*p* = 0.01, Rank-Sum test) between frontal and central cortical lobes. Overall, our behavioral and electrophysiological studies demonstrate a distinguished categorization of pleasant and unpleasant window shapes and more significant ERP modulations in the right than left hemisphere for unpleasant windows compared to pleasant ones.

## Introduction

Behavioral and electrophysiological studies have been regularly used to distinguish the state of brain function of human or non-human subjects with visual stimuli in either conscious or unconscious conditions. Developments of noninvasive technologies in neuroscience have provided excellent opportunities to simultaneously study the activity throughout the cortical lobes. Electroencephalogram (EEG) recording is commonly used in the clinic and research studies for diagnosing neurological disorders such as seizures, as well as measuring actual brain function. It has been shown that the architectural features of a visual scene significantly influence the perception, social behaviors and reactions of human observers to their environments (Delvin and Nasar, 1989; Madani Nejad, 2007; Kamkar et al., 2018), while their underlying neural mechanisms are less well understood (Vartanian et al., 2013; Papale et al., 2016). Therefore, a more detailed understanding of how the brain perceives and processes the features of building components is an interesting goal in neuro-architecture (Chauhan and Moulik, 2014). With evidence-based design perspective, architects and neuroscientists have engaged in a practice to promote human mental state, which focuses on increasing pleasantness in built environments (human-made environments; Papale et al., 2016). Studies have demonstrated a significant relationship between built environments and the human well-being using psychology and physiological indicators of wellness, such as measuring stress, moods, and cognitive performance (Adams, 2013; Cooper et al., 2014; Ghamari and Amor, 2016). Several studies have also demonstrated the significant impact of the built environments, such as the architectural styles (Choo et al., 2017), embodiment (Vecchiato et al., 2015), contours (Vartanian et al., 2013), height and enclosure (Vartanian et al., 2015), lighting and luminance color (Küller et al., 2009; Choi et al., 2014), built vs. natural environment (Sternberg, 2010; Roe et al., 2013; Banaei et al., 2015, 2017) on the subjects’ cortical activity and aesthetic judgments in architectural design. Physical features are the most defining attributes of building facades, which are composed of many visual components. Forms and geometric shapes are the important aspects of visual components in architectural design, and they are one of the most challenging aspects of the design process (Madani Nejad, 2007). In the present study, the effect of geometric window shapes of building facades on cortical activity and emotional reactions was investigated using EEG and psychophysics experiments. Our results demonstrate a distinguished categorization of pleasant and unpleasant window shapes and more significant event related potential (ERP) response modulations in the right hemisphere than the left for unpleasant windows compared to pleasant ones.

## Materials and Methods

### Participants

Eleven graduate students (six females and five males) participated in this experiment. The average age of participants was 26 years old. Two subjects were excluded from analysis because of the high EEG artifacts (e.g., eye-blinks, eye and skin movements) and the remaining nine subjects (four females and five males) were used for the final sample. According to the physiological effects of the menstrual cycles in cognition and emotional expressions (Yamazaki and Tamura, 2017), the female subjects were considered to perform the experimental tasks during their follicular phase (8–14 days of menstrual cycles). All participants had normal vision and none reported a history of neurological disease. At the beginning, each subject received the standard information about the experimental design and procedures and they were also trained for the task before performing the experiment.

All procedures were performed in accordance with the guidelines of the IPM Brain Engineering Research Center and the proposal was approved by the Research Ethics Committee of Institute for Research in Fundamental Sciences (IPM). Written informed consent was obtained from all subjects prior to this experiment.

### Architectural Stimuli and Stimulus Modeling

In the first step, 600 building facades were captured in Gorgan city located in the north of Iran. All pictures were categorized to distinguish architectural parameters like geometry and proportions of window shapes in residential building facades. In the next step, 24 basic building facades were modeled by 3D max software, then 16 building facades among them were selected as the visual stimuli by the Self-Assessment Manikin (SAM) rating test.

### Experimental Design and Procedure

#### The Self-Assessment Manikin (SAM) Rating Test

**Figure 1:**
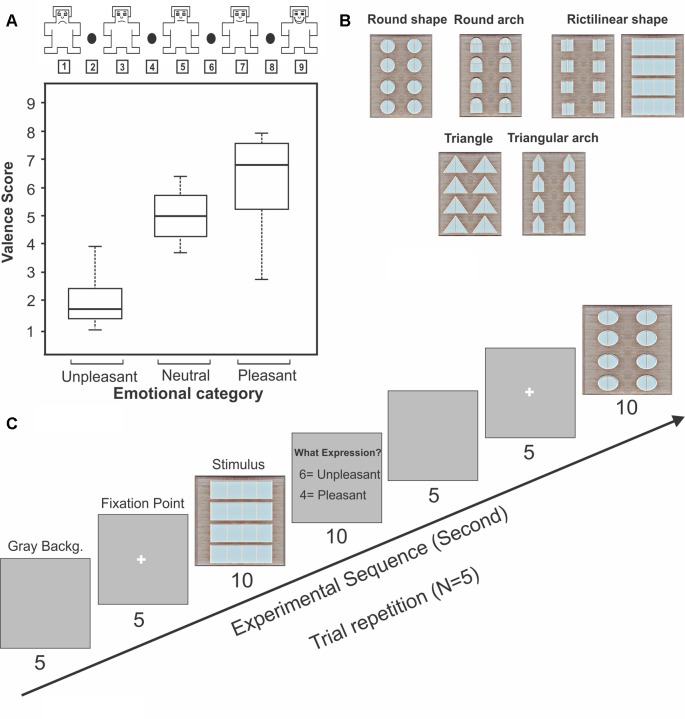
The Self-Assessment Manikin (SAM) rating test and experimental design for electroencephalogram (EEG) recording. Panel **(A)** represents the SAM rating of geometric windows as a function of the emotional categories. The box shows the upper and lower quartiles, the bar inside the box represents the median, and error bars indicate the variability outside the upper and lower quartiles. Panel **(B)** represents the sample of geometric window shapes. Panel **(C)** represents the experimental procedure for EEG recording setup.

The SAM rating test (Bradley and Lang, 1994; Nazari et al., 2012; Geethanjali et al., 2017) was used in the present study for identifying the emotional response of subjects to the geometric window shapes. Participants rated each stimulus according to the pleasant, unpleasant, and neutral emotional state after presenting each stimulus. In the SAM test, each stimulus was rated between 1 and 9 [unpleasant (1–4), neutral (5), pleasant (6–9), Figure 1A]. Subjects were asked to mark the 24 window shapes as neutral, pleasant, or unpleasant stimuli. Then, the only pleasant and unpleasant windows (identified 16 windows by subjects) were used for continuing the EEG recording experiments. Moreover, this behavioral testing procedure was also repeated during the recordings (by pressing the keyboard) to verify the pleasant from the unpleasant stimuli. In sum, subjects identified the 11 window shapes as the pleasant category and the five windows as the unpleasant category.

#### Experimental Procedure for EEG Recording

Subjects sat on a recliner in a sound-attenuated room after arriving at the laboratory. Task instructions were given after placing electrodes on the subject’s head. At the beginning of each session, a gray background was exposed for 5 s then a white fixation point was marked in the center of the screen. After the fixation point, the stimulus in the full size of the monitor was presented for the subjects (10 s, 5 trial repetitions randomly for each stimulus). Subjects were asked to report pleasantness or unpleasantness of each stimulus by pressing the keys, respectively buttons 4 and 6 (Figure 1C). The behavioral results obtained during the recording sessions (by pressing the keys) were finally verified with the SAM test to distinguish the pleasant from the unpleasant stimuli. Figure 1B represents the procedure of task performance.

**Figure 2:**
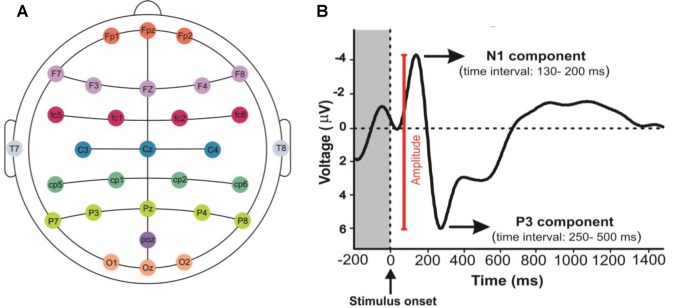
Mapping EEG Electrodes and event related potential (ERP) component signals: **(A)** it shows the position of electrodes on the scalp. **(B)** N1 and P3 components represent the N100 and the P300, respectively. The response amplitude was measured as the voltage from N1 to P3 components.

The visual architectural stimuli were displayed by Psychopy (an open source software) on the acer S221HQLBD LCD monitor (21.5 inch). The computer screen was placed 90 cm in front of the viewer. EEG recording system, ANT Neuro ASA-Lab 64 + 8 ES, was used with 64 channels in this study (only 32 channels used in our study) which the Ag/AgCl electrodes were placed along the scalp according to the international 10–20 system. The electrodes A1 and Fpz were considered as the reference and ground channels, respectively (Figure 2A). Signals were sampled at 16 kHz and the impedance of each electrode was maintained at 5 kΩ or less. The control PC with Psychopy was used for running the codes to synchronize data collection and send event markers to EEG using a parallel port connection.

## Results

### Statistical Data Analysis

The MATLAB (R2012a) was used for preprocessing and filtering neural data and plotting the ERP signals. The EEGlab v13_6_5b toolbox (Topographic maps), R version 3.4.2. Software (Statistical analysis) was applied for plotting and statistical analysis. Friedman test was conducted with MATLAB to assess the behavioral results. Friedman test reported the significance level of differences between two independent variables (pleasant and unpleasant) after calculating the mean of both categories. The results of EEG analysis were assessed in R software by Wilcoxon signed-rank and rank sum test. This non-parametric test reported the differences in ERP recordings.

All electrode channels were preprocessed using a band pass filter (0.1–60 Hz; MATLAB R2012a). Moreover, a Butterworth notch filter (50 Hz), and ADJUST filter (Mognon et al., 2011) were applied to remove the power line noise and artifacts of eye movements and eye blinks from EEG signals. In this study, 200 ms before and 5 s after the stimulus onset were considered to measure cortical activity. The mean voltage of the channels 200 ms pre-stimulus is used as a baseline voltage, and the signal to noise ratios was calculated for each subject by dividing the voltage measurement obtained after the stimulus onset to the baseline activity (200 ms).

Recent studies have shown that the high-resolution measurements of the newly developed EEG instruments are highly useful for exploring the architecture of the brain from the perspective of neuroimaging research studies (Papale et al., 2016). EEG is one of the most popular methods indicating information about the timing (latency) and intensity (amplitude) of stimulus evaluation.

The ERPs, which are time-locked with stimulus events, reflect the time course of neuronal population activity with a resolution of milliseconds (Hillyard and Anllo-Vento, 1998; Patel and Azzam, 2005). Peaks within an ERP can be classified according to their magnitude, timing relative to stimulus onset, polarity, and anatomical site of generation or function reflected by them. For this reason, the ERP signals are frequently analyzed by measuring the response amplitude and latency of the voltages (Picton, 1996; Luck, 2005; Polich, 2007). In the present study, the amplitude of grand average ERPs across all participants and the peaks of P3, N1 and P2 components were calculated, and the amplitudes of signals (peak to peak) were measured in the parietal and occipital cortical lobes (Figure 2B).

#### The N100

The N1 is a well-known component in visual and sensory tasks. It refers to a large negative-going evoked potential, typically peaking approximately between 130 and 200 ms after stimulation in our study. The N1 is evoked by the onset of visual and sensory stimuli and it is also sensitive to visual attention and the emotional content of visual stimuli (Wascher et al., 2009).

#### The P200

The P2 is a positive-going waveform component whose peak varies in latency between 150 and 250 ms after stimulation. It is one of the most prominent features of the visual ERP signals and has been considered an index of affective picture processing (Carretié et al., 2001). Furthermore, it has been shown that the P2 represents some aspects of higher-order perceptual processing, is modulated by attention, and displays a maximal amplitude at the anterior scalp site (Luck and Hillyard, 1994).

#### The P300

The P3 component reflects a positive deflection in voltage with a latency of roughly 250–500 ms after stimulation. Studies have shown that the P300 consists of two subcomponents, the P3a and P3b. The P3a displays a peak latency in the range from 250 to 280 ms with a maximum positive amplitude over frontal and central electrode sites (Squires et al., 1975; Comerchero and Polich, 1999). Whereas, the P3b displays a peak latency around 300 ms on the electrode sites over parietal lobe (Hruby and Marsalek, 2003; Fjell et al., 2007; Polich, 2007; Vafaii et al., 2016) and it represents the processing of conscious discrimination and emotion in humans (Patel and Azzam, 2005; Weinberg and Hajcak, 2010).

### Behavioral Results: Expression of Pleasant and Unpleasant Feeling by Subjects

#### SAM Rating

The subjective ratings (the SAM test) for the stimuli were not normally distributed. Therefore a non-parametric test was performed. The stimuli were categorized in two groups and these were considered as independent variables. Friedman test was conducted to check the presence of significant difference between pleasant and unpleasant (Schalinski et al., 2014). The results of SAM test (five repeated trials) show that building facades including windows with rectangular, square, circular shapes, and semi-circular arches were considered as pleasant pictures (average median of all pleasant stimuli: 6.87; Figure 1A), whereas windows with triangle and triangular arches reported as unpleasant building façades (average median of all unpleasant stimuli: 1.72; Figure 1A). Statistical analysis revealed that there was a significant difference between these two categories (*χ*^2^ = 9, *p*-value = 0.002, Friedman).

#### Behavioral Response During EEG Recording

**Figure 3:**
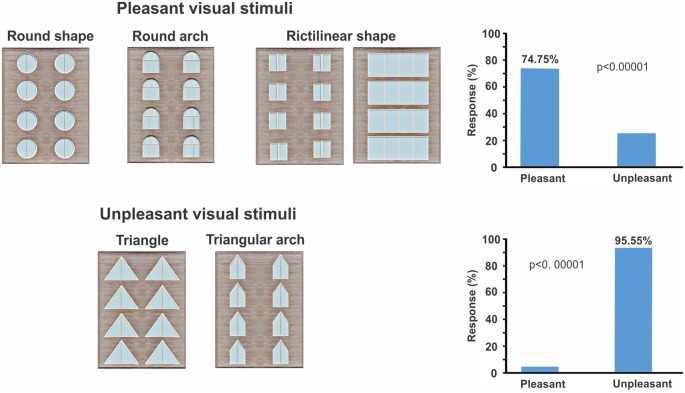
It displays pleasant and unpleasant stimuli based on participants’ viewpoint. Participants were asked to identify pleasant and unpleasant stimuli by the key press (button 4 or 6). The right plot shows the percentage of responses for both pleasant and unpleasant window categories.

The geometric window shapes were distinguished as pleasant and unpleasant windows by subjects. Behavioral results showed that building facades which include windows with rectangular, square, circular shapes, and semi-circular arches were considered pleasant (*p*-value < 0.00001, *z*-score: 5.38 > 1.96; Figure 3), whereas windows with triangle and triangular arches reported as an unpleasant building façade (*p*-value < 0.00001, *z*-score: 6.24 > 1.96; Figure 3).

### Electrophysiological Results: ERP Components

#### Visual Inspection of ERP Signals

The grand average ERP includes some ERP components that are frequently labeled based on polarity, whether the change in voltage is positive or negative in relation to a pre-stimulus baseline. The observed components in this study are the P2, P3 and N1. For the visual N1 and P3 of ERP, the maximal response was observed in the parietal lobe, and for the P2, the maximal response was observed in the frontal lobe.

#### The N100 and P300 ERP Components in Parietal and Occipital Channels

**Figure 4:**
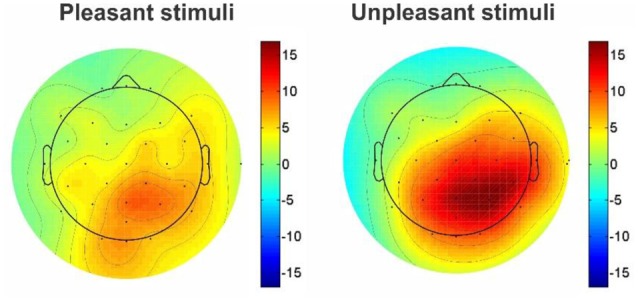
Topographic maps of comparison EEG response activity to the pleasant and unpleasant window shapes.

The mean amplitude of grand average ERPs was investigated by presenting architectural stimuli, geometric window shapes of building facades. Considering N1 and P3 ERP components, a significant difference was reported in the mean amplitude between right hemisphere (electrode channels; p4, p8, o2, cp2, and cp6) and left hemisphere (p7, p3, o1, cp5, and cp1) for both the pleasant pictures [right: 8.085 (μV) and left: 7.249 (μV), *p* < 0.001, Wilcoxon sing rank test] and the unpleasant pictures [right: 8.938 (μV) and left: 7.415 (μV), *p* < 0.001, Wilcoxon sing rank test]. Moreover, the results show that the unpleasant pictures more strongly modulate the right parietal hemisphere than the pleasant ones [Figure 4, the unpleasant = 8.955 (μV) and the pleasant = 8.055 (μV), *p*-value < 0.001].

**Figure 5:**
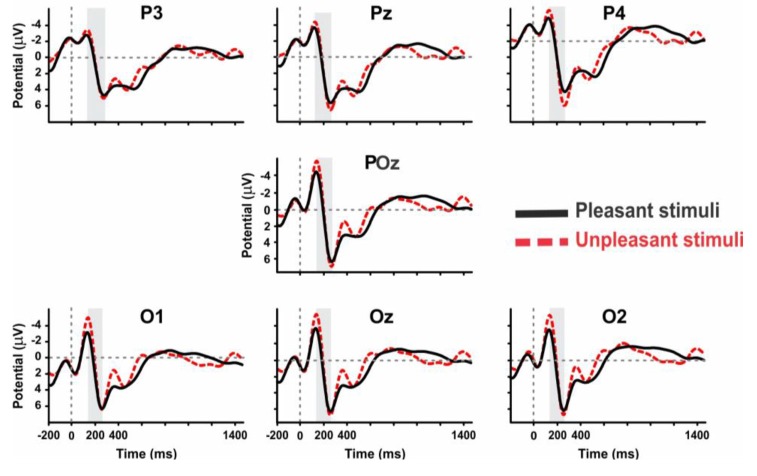
The grand average ERP waveforms of all subjects (*n* = 9) during viewing of pleasant and unpleasant pictures in the posterior region of brain.

Interestingly, we observed that the unpleasant windows show a significantly higher amplitude than pleasant ones in both the parietal [mean amplitude of grand average ERP; unpleasant: 8.83 (μV), pleasant: 7.8 (μV), *p*-value: 0.050, Wilcoxon rank sum test] and the occipital lobes [unpleasant: 9.58 (μV) pleasant: 8.03 (μV), *P* < 0.01, Wilcoxon rank sum test]. Figure 5 demonstrates the ERP amplitude (peak to peak) for both the pleasant and unpleasant stimuli.

**Figure 6:**
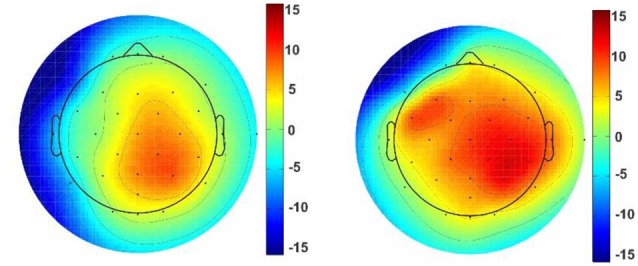
Topographic map of the unpleasant stimuli. Topographic maps reveal ERP voltage (μV) for unpleasant stimuli with triangle (left picture) and triangular arch (right picture) shapes at the p4. The color maps are the averaged amplitude of all unpleasant responses for triangle and triangular arch pictures.

**Figure 7:**
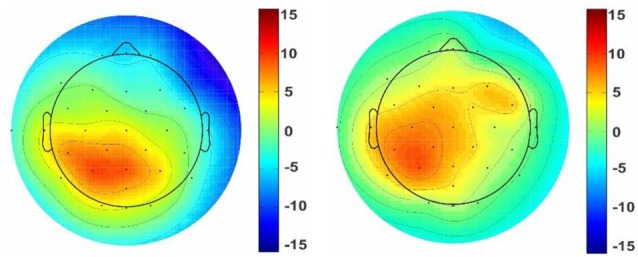
Topographic map of the pleasant stimuli. Topographic maps reveal ERP voltage (μV) for some of the pleasant stimuli such as circle (left picture) and semi-circular arch (right picture) shapes. The color maps are the averaged amplitude of all pleasant responses for circle and semi-circular arch pictures.

For pleasant pictures, the maximum peak (N1, P3) was evoked in the parietal, and for unpleasant pictures, it was evoked in both the parietal and occipital cortical lobes. EEG data analysis also revealed that the peak amplitude of P3 is maximum for the unpleasant stimuli including triangle and triangular arch at the parietal electrode site (p4). In sum, the unpleasant stimuli evoked a significant response in the right hemisphere than the left (Figure 6) while the pleasant stimuli evoked the strong response in the left hemisphere than the right (Figure 7).

#### The P200 ERP Components at the Frontal and Central Lobes

**Figure 8:**
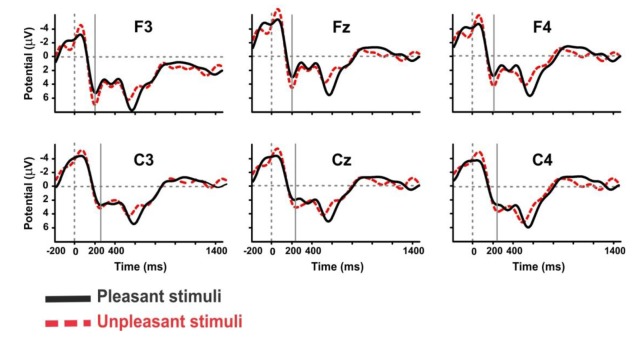
The grand average ERP waveforms of subjects during viewing of pleasant and unpleasant pictures in the anterior area.

The P2 ERP component was also observed in our study. Considering the mean amplitude of the grand average ERP, we report a significant difference between the frontal and central cortical lobes for pleasant pictures (Frontal value: 3.474, Central value: 3.266, *p* = 0.05, Sign Rank Test). For unpleasant stimuli, the mean amplitude of the grand average ERP was also higher in the frontal cortical lobe than the central, but the difference was not significant. Moreover, there was a significant difference in the central cortical lobe between pleasant and unpleasant pictures (Pleasant: 3.266, Unpleasant: 4.421, *p* = 0.01, Rank-Sum Test) and no significant difference in the frontal sites. Observations also revealed a significant difference between pleasant and unpleasant pictures in the anterior (Fz, Cz; *p* < 0.05, Rank-Sum Test), and it was significantly greater for the unpleasant than the pleasant ones (Figure 8). The event of a stimulus-preceding negativity response in the baseline activity in the frontal and occipital channels might be caused either by the perceptual anticipatory activity or by the repeated trials of the stimuli (Brunia et al., 2011; Kotani et al., 2017).

The mean amplitude of grand average ERP in the frontal lobe of the left hemisphere was greater than central lobe, and the mean amplitude in the central of right hemisphere was greater than the frontal lobe, but these were not significant. Furthermore, the anterior amplitude modulations of the left hemisphere were larger for unpleasant picture valence. Finally, the results of this study indicated the mean amplitude was significantly larger for unpleasant pictures valence than the pleasant pictures (*P*-value: 0.024, *p* < 0.05, Rank-Sum Test). The results of this experiment are summarized in Supplementary Table S1.

## Discussion

The present study has investigated the influence of geometrical window shapes of building facades on the emotional states and human cortical activity. To do this, the behavioral response and the signals of EEG recording were measured when the windows were presented to the subjects on the screen in front of them. Our results demonstrated a distinguished categorization of pleasant and unpleasant window shapes as well as their functional lateralization across cortical lobes.

In architecture, the facade of a building is one of the most important aspects for building functionality, the public sphere, and the public health. Our subjects reported that windows with round arch, circular and simple rectilinear shapes represent a pleasant perception, while the triangle or triangular arch window shapes make an unpleasant impression. Our results support the findings of recent studies that have shown that humans prefer and show more pleasant responses toward objects with a curved contour compared to the objects with a sharp-angled contour (Bar and Neta, 2007; Pati et al., 2016). Amir et al. (2011) have also demonstrated that the fMRI activation in the lateral occipital complex, which is an area of the brain associated with shape recognition (James et al., 2003), is greater than that of the straight contours (Amir et al., 2011). It has been also reported that the curvilinear forms in interior residential architectural setting are less stressful than angular and rectangular forms (Madani Nejad, 2007).

On the other hand, one of the most important findings in the present study was a significant relationship between the windows and functional lateralization of cortical activity. It shows that the triangle and triangular arch windows, which were recognized as unpleasant stimuli, evoked the maximum ERP amplitude on the right hemisphere, while round arch and circular shape formats, which were recognized as pleasant windows, evoked the maximum response in the posterior region of the left hemisphere. Several studies have demonstrated that the positive emotional stimuli are lateralized towards the left hemisphere, whereas the negative emotional are lateralized towards the right hemisphere (Reuter-Lorenz and Davidson, 1981; Dimond and Farrington, 1997; Canli et al., 1998; Gainotti, 2011). According to the above findings, we conclude left cortical hemisphere superiority for the pleasant stimuli and right hemisphere superiority for the unpleasant shapes.

Our results also demonstrated that the grand average ERP for the unpleasant stimuli is greater than the pleasant ones. The same result as the large ERP signal for the unpleasant pictures valence has also been reported in a previous study (Olofsson et al., 2008). Furthermore, studies have shown that the negative/unpleasant pictures have more emotional arousal than the positive/pleasant pictures (Cacioppo et al., 1994; Cacioppo and Gardner, 1999; Canli et al., 1998; Öhman and Mineka, 2001; Crawford and Cacioppo, 2002; Keil et al., 2002). Based on the observations and above statements, we can report the peaks of N1, P3 and P2 are maximum for unpleasant pictures valence in the right cortical hemisphere.

Research studies have also demonstrated that the N1 and P3 components are modulated as a function of visual perception and attentional activity in visual cortex (Heinze et al., 1990; Mangun and Hillyard, 1990; Mangun, 1995; Anderer et al., 1996; Hillyard and Anllo-Vento, 1998). The N1 has consistently shown that paying attention to non-spatial object characteristics, such as form and color, modulates ERP signals around 150 ms after the stimulus onset (Hillyard and Anllo-Vento, 1998), while the peak latency of P3 is proportional to stimulus evaluation timing, sensitive to task processing demands, and varies with individual differences in cognitive capability (Polich, 2007). The P2 is thought to index mechanisms of feature detection, stimulus processing (Luck et al., 1994; Jausovec and Jausovec, 2001) selective attention (Hackley et al., 1990; Federmeier and Kutas, 2002) and other early sensory stages (Breznitz, 2008).

Another finding of this study is that the pleasant window shapes evoke maximum ERP amplitude (N1 peak to P3 peak) over parietal, and unpleasant ones created a maximum amplitude in parieto-occipital lobes. As in previous reports, our results also show a maximum amplitude response of the P2 in the frontal electrode sites (Luck et al., 1994; Carretié et al., 2001).

Our results can bolster the previous reports that the affective ERP signals (N1 and P3) revealed maximum amplitude over the parietal cortex (Olofsson et al., 2008). Studies have also demonstrated that the effects of emotional content were the strongest in posterior sites, near occipital-temporal cortex and posterior-parietal cortex (Keil et al., 2002). Moreover, Johnson demonstrated that the component latency of P3 changes across the scalp and is shorter over frontal areas but longer over parietal areas (Johnson, 1993). Importantly, studies have revealed that the P3b appears to occur when subsequent attentional resource activations promote memory operations in temporal-parietal areas (Knight, 1996; Squire and Kandel, 1999; Braázdil et al., 2001, 2003), while the P3b amplitude variation is positively correlated with parietal lobe area (Kayser et al., 1997).

Further, our other findings demonstrate a significant difference between the left and right hemisphere for pleasant and unpleasant window shapes of the building facades. Temporally, ERP amplitude changing patterns of both hemispheres may reflect hemispheric interactions being part of visual picture processing rather than effects of hemispheric specialization for different emotions (Keil et al., 2002). Taken together, the right hemisphere superiorities were prominent for affective pictures. Kayser et al. (1997) had the same belief; they suggested a right-hemispheric superiority for the perception of emotional stimuli, particularly for stimuli with negative valence. However, there is a major strain of evidence, which suggests that both hemispheres process emotionally related behaviors, but do so for different types of emotions. Most commonly, the right hemisphere has been implicated in the regulation of negative effects, while the left is associated with positive emotions (Silberman and Weingartner, 1986).

## Conclusion

The present study investigated the impacts of the architectural features on human cortical activity and emotional state. The findings demonstrated that architectural window shapes have a significant impact on the modulation of cortical activity. In addition, the windows were categorized as pleasant and unpleasant windows by subjects. Essentially, the mean amplitude of ERP signals (peak to peak; P3 to N1) was significantly stronger in both pleasant and unpleasant picture valences in the right hemisphere, particularly in the parietal lobe. However, the unpleasant stimuli show significantly larger amplitude responses than the pleasant ones. Meanwhile, the mean amplitude of the P2 shows a significant difference in the central and frontal lobes for both stimuli, and the unpleasant stimuli show much stronger responses than the pleasant one.

Our findings are also consistent with previous reports, demonstrating that curved contour shapes produce greater FMRI activation in human lateral occipital complex than straight contours do (Amir et al., 2011), and the curvilinear forms in the interior residential architectural setting provide less stress than angular and rectangular forms (Madani Nejad, 2007). We also demonstrated that the effect of pleasant stimuli was larger in the left hemisphere than that of unpleasant ones. According to hemispheric superiority, studies have shown that positive emotions are lateralized towards the left hemisphere, whereas negative emotions are lateralized towards the right hemisphere (Reuter-Lorenz and Davidson, 1981; Dimond and Farrington, 1997; Canli et al., 1998).

## Ethics Statement

All procedures were performed in accordance with the guidelines of the Research Ethics Committee of Institute for Research in Fundamental Sciences (IPM). Written informed consent was obtained from all subjects prior to this experiment.

## Author Contributions

PN, SA, AS, and RL designed and collected data. PN, AS, HS, and RL analyzed the experimental data. PN, AS, and RL wrote the manuscript.

## Conflict of Interest Statement

The authors declare that the research was conducted in the absence of any commercial or financial relationships that could be construed as a potential conflict of interest.

## Supplementary Material

TABLE S1 The mean amplitude of grand average ERP signals for both pleasant and unpleasant picture types.

## References

[B1] Adams M. (2013). Quality of urban space and wellbeing. Int. J. wellbeing 2, 1–21. 10.1002/9781118539415.wbwell064

[B2] Amir O. Biederman I. Hayworth K. J. (2011). The neural basis for shape preferences. Vision Res. 51, 2198–2206. 10.1016/j.visres.2011.08.015 21906615

[B3] Anderer P. Semlitsch H. V. Saletu B. (1996). Multichannel auditory event-related brain potentials: effects of normal aging on the scalp distribution of N1, P2, N2 and P300 latencies and amplitudes. Electroencephalogr. Clin. Neurophysiol. 99, 458–472. 10.1016/s0013-4694(96)96518-9 9020805

[B4] Banaei M. Hatami J. Yazdanfar A. Gramann K. (2017). Walking through architectural spaces: the impact of interior forms on human brain dynamics. Front. Hum. Neurosci. 11:477. 10.3389/fnhum.2017.00477 29033807 PMC5627023

[B5] Banaei M. Yazdanfar A. Nooreddin M. Yoonessi A. (2015). Enhancing urban trails design quality by using electroencephalography device. Procedia Soc. Behav. Sci. 201, 386–396. 10.1016/j.sbspro.2015.08.191

[B6] Bar M. Neta M. (2007). Visual elements of subjective preference modulate amygdala activation. Neuropsychologia 45, 2191–2200. 10.1016/j.neuropsychologia.2007.03.008 17462678 PMC4024389

[B7] Braázdil M. Rector I. Daniel P. Dufek M. Jurak P. (2001). Intracerebral event-related potentials to subthreshold target stimuli. Clinical Neurophysiology. 112, 650–661. 10.1016/s1388-2457(01)00463-1 11275538

[B8] Braázdil M. Roman R. Daniel P. Rektor I. (2003). Intracerebral somatosensory event-related potentials: effect of response type (button pressing versus mental counting) on P3-like potentials within the human brain. Clin. Neurophysiol. 114, 1489–1496. 10.1016/s1388-2457(03)00135-4 12888032

[B9] Bradley M. M. Lang P. J. (1994). Measuring emotion: the self-assessment manikin and the semantic differential. J. Behav. Ther. Exp. Psychiatry 25, 49–59. 10.1016/0005-7916(94)90063-9 7962581

[B10] Breznitz Z. (2008). Brain Research in Language. Berlin: Springer Science+Business Media, LLC.

[B20] Brunia C. H. M. van Boxtel G. J. M. Böcker K. B. E. (2011). “Negative slow waves as indices of anticipation: the Bereitschaftspotential, the contingent negative variation, and the stimulus-preceding negativity,” in The Oxford Handbook of Event-Related Potential Components, eds Kappenman E. S. Luck S. J. (Oxford: Oxford University Press), 189–207. 10.093/oxfordhb/9780195374148.013.0108

[B11] Cacioppo J. T. Crites S. Gardner W. Berntson G. (1994). Bioelectrical echoes from evaluative categorizations I: a late positive brain potential that varies as a function of trait negativity and extremity. J. Pers. Soc. Psychol. 67, 115–125. 10.1037/0022-3514.67.1.115 8046583

[B12] Cacioppo J. T. Gardner W. L. (1999). Emotion. Annu. Rev. Psychol. 50, 191–214. 10.1146/annurev.psych.50.1.191 10074678

[B13] Canli T. Desmond J. E. Zhao Z. Glover G. Gabrieli J. D. E. (1998). Hemispheric asymmetry for emotional stimuli detected with fMRI. Neuroreport. 9, 3233–3239. 10.1097/00001756-199810050-00019 9831457

[B14] Carretié L. Martín-Loeches M. Hinojosa J. A. Mercado F. (2001). Emotion and attention interaction studied through event-related potentials. J. Cogn. Neurosci. 13, 1109–1128. 10.1162/089892901753294400 11784449

[B15] Chauhan R. Moulik A. (2014). Translational science model for designing of outdoor environments for stress relief in an educational institute. Int. J. Innov. Res. Sci. Eng. Technology 3, 17916–17936. 10.15680/ijirset.2014.0312024

[B16] Choi K. M. Jang K. M. Jang K. I. Um Y. H. Kim M. S. Kim D. W.. (2014). The effects of 3 weeks of rTMS treatment on P200 amplitude in patients with depression. Neurosci. Lett. 577, 22–27. 10.1016/j.neulet.2014.06.003 24928222

[B17] Choo H. Nasar J. Nikrahei B. Walther D. B. (2017). Neural codes of seeing architectural styles. Sci. Rep. 7:40201. 10.1038/srep40201 28071765 PMC5223202

[B18] Comerchero M. D. Polich J. (1999). P3a and P3b from typical auditory and visual stimuli. Clin. Neurophysiol. 110, 24–30. 10.1016/s0168-5597(98)00033-1 10348317

[B19] Cooper R. Burton E. Cooper C. (2014). Wellbeing: A Complete Reference Guide, Wellbeing and the Environment. Chichester: Wiley-Blackwel.

[B21] Crawford L. E. Cacioppo J. T. (2002). Learning where to look for danger: integrating affective and spatial information. Psychol. Sci. 13, 449–453. 10.1111/1467-9280.00479 12219812

[B22] Delvin K. Nasar J. L. (1989). The beauty and the beast: some preliminary comparisons of high versus popular residential architecture and public versus architect judgments of same. J. Environ. Psychol. 9, 333–344. 10.1016/s0272-4944(89)80013-1

[B23] Dimond S. J. Farrington L. (1997). Emotional response to films shown to the right or left hemisphere of the brain measured by heart rat. Acta Psychol. 41, 255–260. 10.1016/0001-6918(77)90020-8 883511

[B24] Federmeier K. D. Kutas M. (2002). Picture the difference: electrophysiological investigations of picture processing in the two cerebral hemispheres. Neuropsychologia 40, 730–747. 10.1016/s0028-3932(01)00193-2 11900725

[B25] Fjell A. M. Walhoved B. K. Fischl B. Reinvang I. (2007). Cognitive function, P3a/P3b brain potentials and cortical thickness in aging. Hum. Brain Mapp. 28, 1098–1116. 10.1002/hbm.20335 17370342 PMC6871485

[B26] Gainotti G. (2011). Unconscious processing of emotions and the right hemisphere. Neuropsychologia 50, 205–218. 10.1016/j.neuropsychologia.2011.12.005 22197572

[B27] Geethanjali B. Adalarasu K. Hemapraba A. Pravin Kumar S. Rajasekeran R. (2017). Emotion analysis using SAM (Self-Assessment Manikin) scale. Biomed. Res. 28, 18–24.

[B28] Ghamari H. Amor C. (2016). The role of color in healthcare environments, emergent bodies of evidence-based design approach. Sociol. Anthropol. 4, 1020–1029. 10.13189/sa.2016.041109

[B29] Hackley S. Woldorff M. Hilyard S. (1990). Cross-modal selective attention effects on retinal, myogenic, brainstem and cerebral evoked potentials. Psychophysiology 27, 195–208. 10.1111/j.1469-8986.1990.tb00370.x 2247550

[B30] Heinze H. J. Mangun G. R. Hillyard S. A. (1990). “Visual event-related potentials index perceptual accuracy during spatial attention to bilateral stimuli,” in Psychophysiological Brain Research, eds Brunia C. Gallard A. Kok A. (Netherlands: Tilburg University Press), 196–202.

[B31] Hillyard S. A. Anllo-Vento L. (1998). Event-related brain potentials in the study of visual selective attention. Proc. Natl. Acad. Sci. U S A 95, 781–787. 10.1073/pnas.95.3.781 9448241 PMC33798

[B32] Hruby T. Marsalek P. (2003). Event-related potentials—the P3 wave. Acta Neurobiol. Exp. Wars. 63, 55–63. 12784933 10.55782/ane-2003-1455

[B33] James T. W. Culham J. Humphrey G. K. Milner A. D. Goodale M. A. (2003). Ventral occipital lesions impair object recognition but not object-directed grasping: an fMRI study. Brain. 126, 2463–2475. 10.1093/brain/awg248 14506065

[B34] Jausovec N. Jausovec K. (2001). Differences in EEG current density related to intelligence. Cogn. Brain Res. 12, 55–60. 10.1016/s0926-6410(01)00029-5 11489609

[B35] Johnson R. (1993). On the neural generators of the P300 component of the event-related potential. Psychophysiology 30, 90–97. 10.1111/j.1469-8986.1993.tb03208.x 8416066

[B3900] Kamkar S. H. Moghaddam H. A. Lashgari L. (2018). Early visual processing of feature saliency tasks: a review of psychophysical experiments. Front. Syst. Neurosci. 12:54 10.3389/fnsys.2018.00054 30416433 PMC6212481

[B36] Kayser J. Tenke C. Nordby H. Hammerborg D. Hugdahl K. Erdmann G. (1997). Event-related potential (ERP) asymmetries to emotional stimuli in a visual half-field paradigm. Psychophysiology 34, 414–426. 10.1111/j.1469-8986.1997.tb02385.x 9260494

[B37] Keil A. Bradley M. Hauk O. Rockstroh B. Elbert T. Lang P. (2002). Large-scale neural correlates of affective picture processing. Psychophysiology 39, 641–649. 10.1111/1469-8986.3950641 12236331

[B38] Knight R. T. (1996). Contribution of human hippocampal region to novelty detection. Nature 383, 256–259. 10.1038/383256a0 8805701

[B39] Kotani Y. Ohgami Y. Yoshida N. Kiryu S. H. Inoue Y. (2017). Anticipation process of the human brain measured by stimulus-preceding negativity (SPN). J. Phys. Fit. Sports Med. 6, 7–14. 10.7600/jpfsm.6.7

[B40] Küller R. Mikellides B. Janssens J. (2009). Color, arousal and performance—A comparison of three experiments. Color Res. Appl. 34, 141–152. 10.1002/col.20476

[B42] Luck S. J. (2005). “Ten simple rules for designing and interpreting ERP experiments,” in Event-Related Potential: A Method Handbook, ed. Handy T. C. (Cambridge, MA: The MIT Press), 17–32.

[B41] Luck S. J. Hillyard S. A. (1994). Electrophysiological correlates of feature analysis during visual search. Psychophysiology 31, 291–308. 10.1111/j.1469-8986.1994.tb02218.x 8008793

[B43] Luck S. J. Hillyard S. A. Mouloua M. Woldorff M. G. Clark V. P. Hawkins H. L. (1994). Effects of spatial cuing on luminance detectability: psychophysical and electrophysiological evidence for early selection. J. Exp. Psychol. Hum. Percept. Perform. 20, 887–904. 10.1037/0096-1523.20.4.887 8083642

[B44] Madani Nejad K. (2007). Curvilinearity in Architecture: Emotional Effect of Curvilinear Forms in Interior Design. Texas, TX: Texas A&M University. Doctoral Dissertation Available online at: http://hdl.handle.net/1969.1/5750

[B45] Mangun G. R. (1995). Neural mechanisms of visual selective attention. Psychophysiology. 32, 4–18. 10.1111/j.1469-8986.1995.tb03400.x 7878167

[B46] Mangun G. R. Hillyard S. A. (1990). Allocation of visual attention to spatial locations: tradeoff functions for event-related brain potentials and detection performance. Percept. Psychophys. 47, 532–550. 10.3758/bf03203106 2367174

[B47] Mognon A. Jovicich J. Bruzzone L. B uiatti M. (2011). ADJUST: an automatic EEG artifact detector based on the joint use of spatial and temporal features. Psychophysiology 48, 229–240. 10.1111/j.1469-8986.2010.01061.x 20636297

[B48] Nazari M. N. Nabizadeh Chianeh G. H. Vahedi S. H. Rostami M. (2012). Validity and reliability of self-assessment manikin. Res. Psychol. Health 6, 52–61.

[B49] Öhman A. Mineka S. (2001). Fears, phobias and preparedness: toward an evolved module of fear and fear learning. Psychol. Rev. 108, 483–522. 10.1037/0033-295x.108.3.483 11488376

[B50] Olofsson J. K. Nordin S. Sequeira H. Polich J. (2008). Affective picture processing: an integrative review of ERP finding. Biological Psychology. 77, 247–265. 10.1016/j.biopsycho.2007.11.006 18164800 PMC2443061

[B51] Papale P. Chiesi L. Rampinini A. C. Pietrini P. Ricciardi E. (2016). When neuroscience touches architecture: from hapticity to a supramodel functioning of the human brain. Front. Psychol. 7:866. 10.3389/fpsyg.2016.00866 27375542 PMC4899444

[B52] Patel S. H. Azzam P. N. (2005). Characterization of N200 and P300: selected studies of the event-related potential. Int. J. Med. Sci. 2, 147–154. 10.7150/ijms.2.147 16239953 PMC1252727

[B53] Pati D. O’Boyle M. Hou J. Nanda U. Ghamari H. (2016). Can hospital form trigger fear response? HERD 9, 1–14. 10.1177/1937586715624210 26747839

[B54] Picton T. W. (1996). Electrophysiology of mind: event-related brain potentials and cognition. Edited by Michael D. Rugg and Michael G.H. Coles. Oxford, England: Oxford University Press, 1995. Psychophysiology 33, 612–613. 10.1111/j.1469-8986.1996.tb02439.x

[B55] Polich J. (2007). Updating P300: an integrative theory of P3a and P3b. Clin. Neurophysiol. 118, 2128–2148. 10.1016/j.clinph.2007.04.019 17573239 PMC2715154

[B56] Reuter-Lorenz P. Davidson R. J. (1981). Differential contributions of the two cerebral hemispheres to the perception of happy and sad faces. Neuropsychologia 19, 609–613. 10.1016/0028-3932(81)90030-0 7279195

[B57] Roe J. J. Aspinall P. A. Mavros P. Coyne R. (2013). Engaging the brain: the impact of natural versus urban scenes using novel EEG methods in an experimental setting. Environ. Sci. 1, 93–104. 10.12988/es.2013.3109

[B58] Schalinski I. Moran J. Schauer M. Elbert T. H. (2014). Rapid emotional processing in relation to trauma-related symptoms as revealed by magnetic source imaging. BMC Psychiatry 14:193. 10.1186/1471-244x-14-193 24997778 PMC4100056

[B59] Silberman E. K. Weingartner H. (1986). Hemispheric lateralization of functions related to emotion. Brain Cogn. 5, 322–353. 10.1016/0278-2626(86)90035-7 3530287

[B60] Squire L. Kandel E. (1999). Memory From Mind to Molecules. New York, NY: Scientific American Library.

[B61] Squires N. K. Squires K. C. Hillyard S. A. (1975). Two varieties of long-latency positive waves evoked by unpredictable auditory stimuli in man. Electroencephalogr. Clin. Neurophysiol. 38, 387–401. 10.1016/0013-4694(75)90263-1 46819

[B62] Sternberg E. M. (2010). Healing Spaces: The Science of Place and Well-Being. Cambridge, MA: Harvard University Press.

[B63] Vafaii P. Mazhari S. H. Pourrahimi A. M. Nakhee N. (2016). Hemispheric differences for visual P3 amplitude in patients with schizophrenia. Neuropsychiatry 6, 308–313. 10.4172/neuropsychiatry.1000154

[B64] Vartanian O. Navarrete G. Chatterjee A. Fich L. B. Gonzalez-Mora J. L. Leder H. (2015). Architectural design and the brain: effects of ceiling height and perceived enclosure on beauty judgments and approach avoidance decisions. J. Environ. Psychol. 41, 10–18. 10.1016/j.jenvp.2014.11.006

[B65] Vartanian O. Navarrete G. Chatterjee A. Fich L. B. Leder H. Modroño C.. (2013). Impact of contour on aesthetic judgments and approach-avoidance decisions in architecture. Proc. Natl. Acad. Sci. U S A 110, 10446–10453. 10.1073/pnas.1301227110 23754408 PMC3690611

[B66] Vecchiato G. Tieri G. Jelic A. De Matteis F. Maglione A. G. Babiloni F. (2015). Electroencephalographic correlates of sensorimotor integration and embodiment during the appreciation of virtual architectural environments. Front. Psychol. 6:1944. 10.3389/fpsyg.2015.01944 26733924 PMC4686624

[B67] Wascher E. Hoffmann S. Sanger J. Grosjean M. (2009). Visuo-spatial processing and the N1 component of the ERP. Psychophysiology 46, 1270–1277. 10.1111/j.1469-8986.2009.00874.x 19744158

[B68] Weinberg A. Hajcak G. (2010). Beyond good and evil: the time-course of neural activity elicited by specific picture content. Emotion 10, 767–782. 10.1037/a0020242 21058848

[B69] Yamazaki M. Tamura K. (2017). The menstrual cycle affects recognition of emotional expressions: an event-related potential study. F1000Res. 6:853. 10.12688/f1000research.11563.1 28868136 PMC5558101

