# Supplementary material for: Encoding Pleasant and Unpleasant Expression of the Architectural Window Shapes: An ERP Study
Source: Front Behav Neurosci. 2019 Aug 16;13:186. doi: 10.3389/fnbeh.2019.00186 (PMC6707382; doi:10.3389/fnbeh.2019.00186)
Supplement: TABLE S1 — The mean amplitude of grand average ERP signals for both pleasant and unpleasant picture types. [file Table_1.DOCX]

| **Category** | **Electrode sites** | | | | | | | | | | | | | |
| --- | --- | --- | --- | --- | --- | --- | --- | --- | --- | --- | --- | --- | --- | --- |
|  | **Frontal** | | | **Central** | | | **Parietal** | | | | **Occipital** | | | |
|  | **F4** | **Fz** | **F3** | **C4** | **Cz** | **C3** | **p4** | **Pz** | | **p3** | **O2** | **Oz** | | **O1** |
|  | P2 mean amplitude (μv) | | | | | | The mean amplitude of P3 to N1 components (peak to peak μv) | | | | | | | |
| **Pleas.** | 3.43 ± 0.68 | 2.99± 0.67 | 3.56± 0.64 | 3.25 ± 0.71 | 3.12 ± 0.74 | 3.42 ± 0.64 | 9.75 ± 0.02 | | 10.26 ± 0.03 | 7.71 ± 0.1 | 8.17 ± 0.24 | | 8.46 ± 0.26 | 7.47 ± 0.29 |
| **Unpleas.** | 3.92 ± 0.98 | 4.63±0.99 | 4.13± 0.95 | 4.682 ± 0.88 | 4.341± 0.87 | 4.20 ± 0.85 | 10.59± 0.17 | | 11.24± 0.17 | 8.14 ± 0.16 | 9.93 ± 0.17 | | 10.01 ± 0.20 | 8.80 ± 0.25 |
| **pvalue** | NS | 0.009 | NS | 0.01 | 0.02 | NS | 0.03 | | NS | NS | 0.007 | | 0.01 | NS |
